# On the nature of the plant ER exit sites

**DOI:** 10.3389/fpls.2022.1010569

**Published:** 2022-10-07

**Authors:** Alastair J. McGinness, Jennifer Schoberer, Charlotte Pain, Federica Brandizzi, Verena Kriechbaumer

**Affiliations:** ^1^ Endomembrane Structure and Function Research Group, Department of Biological and Medical Sciences, Oxford Brookes University, Oxford, United Kingdom; ^2^ Department of Applied Genetics and Cell Biology, Institute of Plant Biotechnology and Cell Biology, University of Natural Resources and Life Sciences, Vienna, Austria; ^3^ MSU-DOE Plant Research Lab, Michigan State University, East Lansing, MI, United States; ^4^ Great Lakes Bioenergy Research Center, Michigan State University, East Lansing, MI, United States; ^5^ Department of Plant Biology, Michigan State University, East Lansing, MI, United States

**Keywords:** endoplasmic reticulum, Golgi body, exit site, ERES, Sec16, Sar1a, Sec24, ER exit sites

## Abstract

In plants, the endoplasmic reticulum (ER) and Golgi bodies are not only in close proximity, but are also physically linked. This unique organization raises questions about the nature of the transport vectors carrying cargo between the two organelles. Same as in metazoan and yeast cells, it was suggested that cargo is transported from the ER to Golgi cisternae *via* COPII-coated vesicles produced at ribosome-free ER exit sites (ERES). Recent developments in mammalian cell research suggest, though, that COPII helps to select secretory cargo, but does not coat the carriers leaving the ER. Furthermore, it was shown that mammalian ERES expand into a tubular network containing secretory cargo, but no COPII components. Because of the close association of the ER and Golgi bodies in plant cells, it was previously proposed that ERES and the Golgi comprise a secretory unit that travels over or with a motile ER membrane. In this study, we aimed to explore the nature of ERES in plant cells and took advantage of high-resolution confocal microscopy and imaged ERES labelled with canonical markers (Sar1a, Sec16, Sec24). We found that ERES are dynamically connected to Golgi bodies and most likely represent pre-*cis*-Golgi cisternae. Furthermore, we showed fine tubular connections from the ER to Golgi compartments (ERGo tubules) as well as fine protrusions from ERES/Golgi cisternae connecting with the ER. We suggest that these tubules observed between the ER and Golgi as well as between the ER and ERES are involved in stabilizing the physical connection between ER and ERES/Golgi cisternae, but may also be involved in cargo transport from the ER to Golgi bodies.

## Introduction

In plants, the Golgi apparatus (GA) comprises many distinct stacks of membrane-bounded cisternae, approximately 1 micron in diameter that tend to be distributed around the cytoplasm. In many cell types, especially highly vacuolated cells, Golgi stacks are motile (Boevink et al., 1998) and closely associated with the endoplasmic reticulum (ER). This close association has been demonstrated by the application of optical laser tweezers, which can trap individual Golgi bodies and pull strands of attached ER behind them (Sparkes et al., 2009). Attachment to the ER however may not occur *via* membrane connections, such as hemifusion, as it has been shown that AtCASP, a member of the golgin family of proteins, may act as a proteinaceous tether between the ER and Golgi (Osterrieder et al., 2017). Due to this very close association between the ER and Golgi bodies, it was proposed that the cargo exit sites on the ER surface (ER exit sites, ERES) and the Golgi comprise a secretory unit that travels over or with a motile ER membrane (daSilva et al., 2004). The close association between the ER and Golgi bodies raises the question of the nature of the transport vectors carrying cargo between the two organelles.

It is generally accepted that most cargo from the ER reaches the Golgi *via* mechanisms dependent on the COPII machinery (for recent reviews see (Brandizzi, 2018; Kurokawa and Nakano, 2019; Robinson, 2020; Kriechbaumer and Brandizzi, 2020). COPII proteins are a set of functionally conserved proteins such as the cytosolic small GTPase Sar1, the ER membrane protein guanine nucleotide exchange factor (GEF) Sec12 and components of the COPII coat Sec23/24 and Sec13/31. Retrograde transport back from the Golgi bodies to the ER well as retrograde transport within the Golgi apparatus is facilitated by the COPI machinery (Oprins et al., 1993; Arakel and Schwappach, 2018; Robinson, 2020). COPI vesicles were identified by immunogold labelling of cryosections using antibody-labelling of COPI components such as the GTPase ARF1 and different coatomer subunits (Orci et al., 2000; Pimpl et al., 2000; Donohoe et al., 2007; Langhans et al., 2012) but COPII vesicles have been rarely observed (Langhans et al., 2012), raising the question on the identity of the carriers operating in ER-to-Golgi transport.

Indeed, it appears that several types of carriers (tubules, saccules, tunnels, and coated vesicles) may coexist and operate along the ER-to-Golgi route allowing for a diverse, flexible and robust trafficking system. For instance in mammalian cells, using 3D electron-tomography (Zeuschner et al., 2006) it was shown that ERES are continuous with the ER and capable of adapting in size depending on the secretory load (Farhan et al., 2008; Boncompain et al., 2012; Weigel et al., 2021; Shomron et al., 2021). It was previously suggested by the metazoan and yeast research communities that the anterograde vectors for general cargo are COPII-coated vesicles produced at ribosome-free ERES and COPII coated tubules could carry larger proteins (Gorur et al., 2017). COPII vesicles and tubules would then deliver cargo directly to the *cis*-Golgi in yeast (Matsuura-Tokita et al., 2006) or to an intermediate vesiculo-tubular compartment that is transported over varying distances to the *cis*-Golgi in animal cells (Tang et al., 2005). However, various reports suggested that ER-to-Golgi transport requires both vesicular and tubular compartments to sort the ERES cargo to the Golgi apparatus (Saraste and Svensson, 1991; Bannykh et al., 1996; Xu and Hay, 2004; Watson and Stephens, 2005). Assays with temperature-sensitive fluorescently tagged cargo also showed that cargo colocalizes with COPII components at the ERES but does not exit the ERES with the COPII coat component Sec24, suggesting that at least parts of Golgi trafficking might be COPII independent (Presley et al., 1997; Stephens et al., 2000). Indeed, it was reported that COPII components remain on the ERES and that cargo is transported in Rab1-dependent carriers that are not labelled by COPII proteins (Westrate et al., 2020). These data highlight that vesicle carriers are not the only means for anterograde transport and highlight the possibility that COPII is rather involved in secretory cargo selection than its actual transport.

In support of this, recent data in mammalian cells showed that ERES expand into a tubular network containing secretory cargo but no COPII components. COPII components were found to decorate the neck of these tubules, implicating that the departing transport carrier is not coated (Weigel et al., 2021; Shomron et al., 2021). Based on the findings that COPII does not coat the carriers leaving the ER, it was suggested that COPII helps solely to select secretory cargo.

The plant trafficking community has generally embraced the concept of COPII vesicle vectors, due to the presence of all the structural proteins required for COPII coat construction (Kang and Staehelin, 2008). However, there is little microscopy evidence for the existence of such vesicles (Langhans et al., 2012), and due to the limited space between the ER and *cis*-Golgi an alternative hypothesis based on direct tubular connections between the two organelles has been proposed and debated over the years (Hawes et al., 2008; Hawes, 2012; Robinson et al., 2015; Kriechbaumer and Brandizzi, 2020).

Here we show that by utilizing high-resolution microscopy in live plant cells and by considering geometry of the donor and acceptor membranes, ERES are associated with the *cis*-face of Golgi cisternae rather than associated with the ER surface. Moreover, multiple small ER-derived tubules are associated with most Golgi cisternae. Thus, in plants, the nature of the ER-Golgi interface and even the nature of ERES in their currently accepted form should be re-evaluated.

## Materials and methods

### Plant growth and Agrobacterium-mediated transient protein expression

*Nicotiana tabacum* (SR1 cv Petit Havana) plants were grown for transient Agrobacterium-mediated expression, as previously reported (Sparkes et al., 2006). In brief, transformed agrobacteria were pelleted by centrifugation at 1800 g at room temperature for 5 min. Infiltration buffer (5 mg ml^−1^ glucose, 50 mM MES, 2 mM Na_3_PO_4_·12H_2_O and 0.1 mM acetosyringone) was used to wash the pellet once and then to resuspend the agrobacteria in 1 ml. The bacterial suspension was diluted in the infiltration buffer to an OD_600_ of 0.1. The final dilution of the infiltration medium was injected through the stomata on the underside of the tobacco leaf using a 1 ml syringe. Infiltrated plants were kept at 22°C for 72 hours prior to imaging.

*Arabidopsis thaliana* (Col-0) plants expressing GFP-HDEL and ST-mRFP in a stable manner were briefly sterilized with 70% ethanol and placed on plates with ½ strength Murashige and Skoog medium. The plates were stratified at 4°C in the dark for 3 days before being transferred to a plant incubator at 22°C with 16 hours light and 8 hours dark.

### Confocal microscopy

Transformed leaf epidermal samples were imaged using a Zeiss PlanApo ×100/1.46 NA oil immersion objective on a Zeiss LSM880 confocal equipped with Airyscan detector. Typically 512 × 512 images and time-lapse sequences were collected in 8-bit with 2-line averaging at an (x,y) pixel spacing of 20–80 nm with excitation at 488 nm (GFP) and 561 nm (RFP), and emission at 495–550 nm and 570–615 nm, respectively.

Distances between markers were analyzed using Zen line profiles, measuring the distance between the maximum of the corresponding fluorescence peaks. Significance was calculated by Kruskal-Wallis (*p< 0.05; **p< 0.01; ***p< 0.001).

### Drug treatments

Segments (roughly 5 mm^2^) of transformed leaves were used for drug treatment, confocal imaging, and analysis. For inhibition of actin polymerization, leaf tissue was submerged in 25 µM latrunculin B (stock solution: 10 mM in DMSO) for 30 mins. BFA (stock solution: 10 mg ml^-1^ in DMSO) was used at a concentration of 100 µg ml^-1^ (Brandizzi et al., 2002). BFA washes were performed as described earlier (Brandizzi et al., 2002). All stock solutions were kept at -20°C, working solutions were prepared fresh just before use.

## Results

### High-resolution imaging of the ER-Golgi interface in live plant cells

To obtain high-resolution imaging, we implemented a Zeiss LSM880 confocal microscope equipped with Airyscan detector, which enables up to 120 nm lateral resolution. We imaged cells coexpressing markers for the ER lumen (GFP-HDEL; (Brandizzi et al., 2003)) and the medial/*trans* cisternae of Golgi bodies (sialyltransferase (ST)-mRFP; (Runions et al., 2006)). Proteins were first expressed transiently in tobacco leaf epidermal cells (Sparkes et al., 2006).

**Figure 1:**
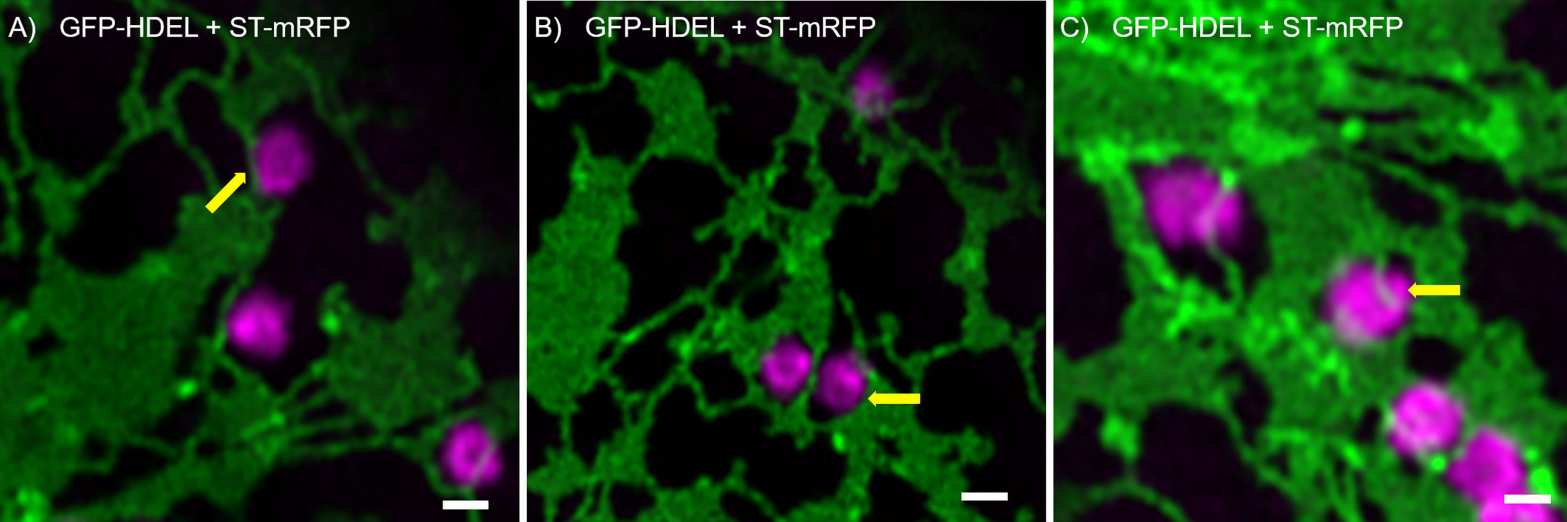
ER and Golgi are in close association as demonstrated by high-resolution microscopy. The ER marker GFP-HDEL (in green) and the Golgi body marker ST-mRFP (in magenta) are transiently expressed in tobacco leaf epidermal cells. Golgi bodies are associated with ER tubules **(A)**, yellow arrow) and cisternal edges (**B**, yellow arrow) and can sit in small lacunae within ER cisternae (**C**, white arrow). Size bars = 1 µm.

We found that Golgi bodies are motile and associated with ER tubules (**Figure 1A**; **Supplementary movie S1**) or curved edges of cisternae (**Figure 1B**) or may be trapped within small lacunae in ER cisternae (**Figure 1C**). Although connections between ER and Golgi bodies have been described before (daSilva et al., 2004; Sparkes et al., 2009; Osterrieder et al., 2017; Vieira et al., 2020), our results indicate that we can visualize the spatial distribution of the ER and Golgi in live cells at a level of resolution that has not been reported before.

### Fine dynamic tubules connect ER and Golgi bodies

**Figure 2:**
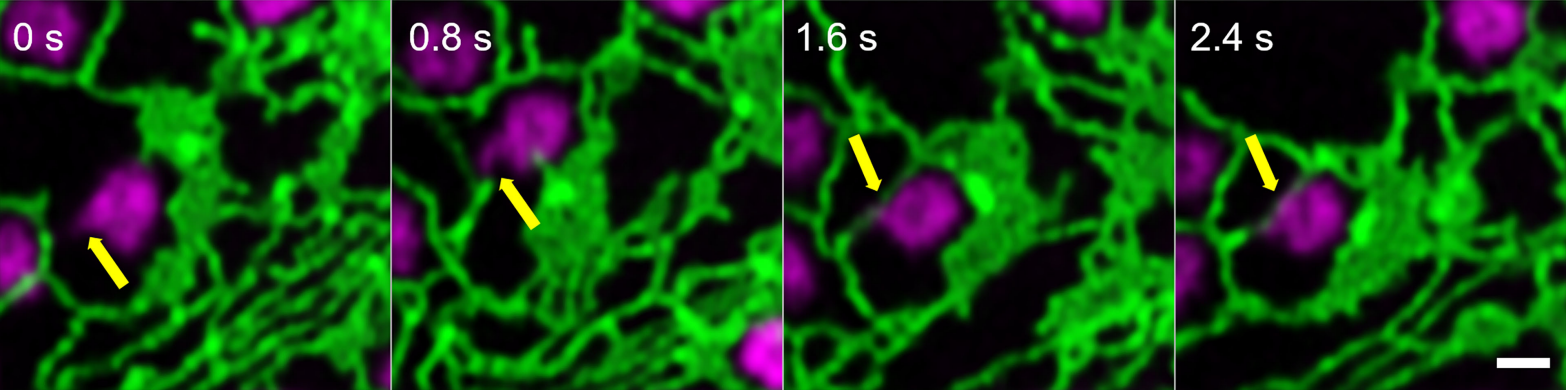
Identification of dynamic Golgi-derived tubules that connect with the ER. Golgi bodies labelled with the medial/*trans*-Golgi marker ST-mRFP (in magenta) form protrusions (yellow arrow) that can also be wrapped around ER tubules (labelled with GFP-HDEL in green). Markers are expressed transiently in tobacco leaf epidermal cells. Images are shown in time intervals of 0.8 s between the frames and time points are indicated. Size bar = 1 µm.

Looking at the dynamics and the shaping of Golgi bodies with high-resolution live-cell microscopy, we found that all Golgi bodies visualized produce small membrane protrusions from their surface that are very dynamic but always make contact with or are wrapped around ER tubules (**Figure 2**), allowing for a constant physical but highly dynamic connection between the two organelles.

**Figure 3:**
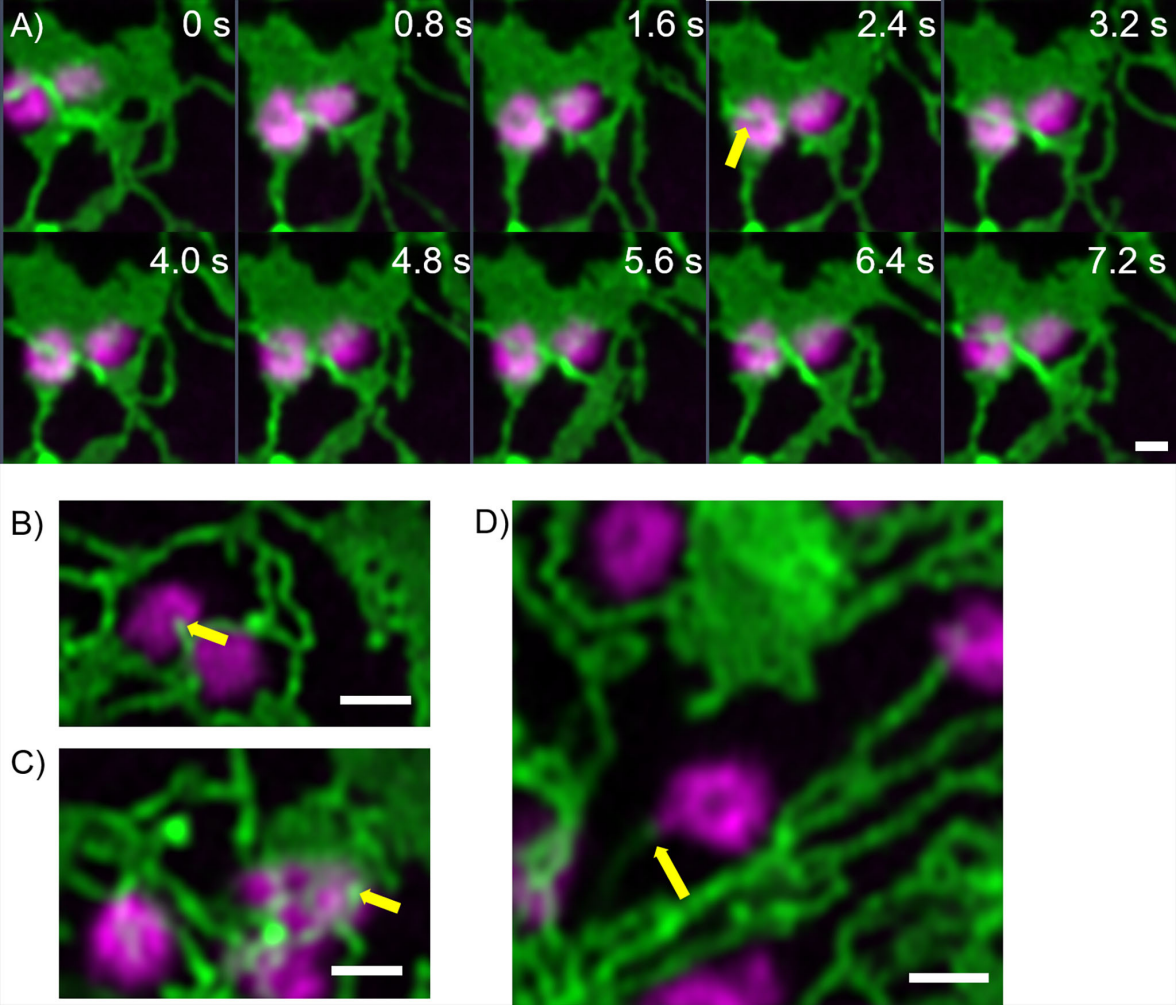
Uniquely shaped ER tubules connect with Golgi bodies. Fine ER tubules thread through (**A, B** yellow arrows) and around the Golgi bodies (**C** yellow arrow) and can connect with Golgi protrusions (**D**, yellow arrow). Golgi bodies are labelled with the medial/*trans*-Golgi marker ST-mRFP (in magenta) and the ER is labelled with GFP-HDEL in green. Markers are expressed transiently in tobacco leaf epidermal cells. Images for **A**) are shown in time intervals of 0.8 s between the frames and time points are indicated. Size bars = 1 µm.

**Figure 4:**
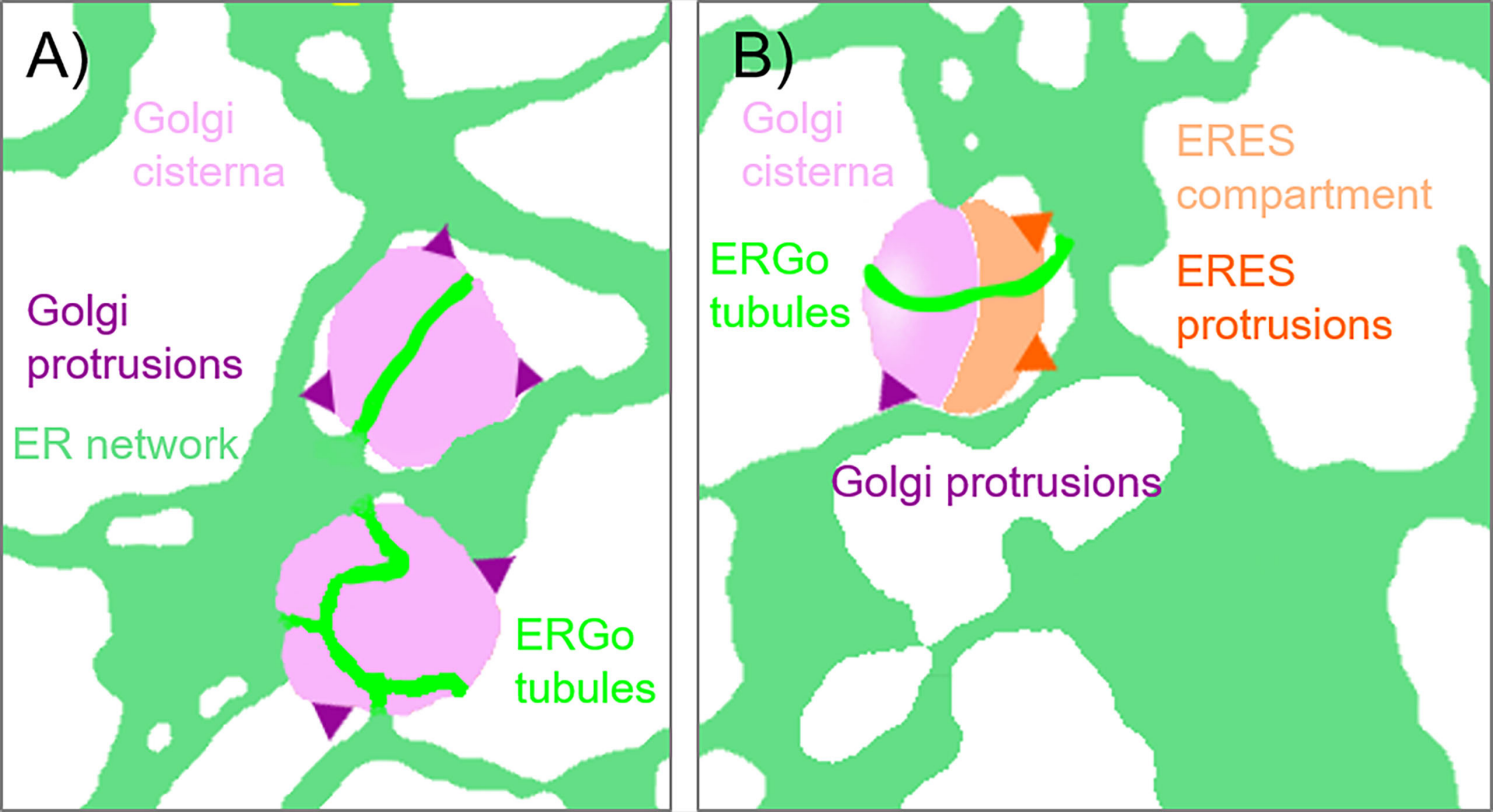
Schematic representation of ER-Golgi connections. The diagram indicates the various connections observed between ER and Golgi bodies: **(A)** ERGo tubules (dark green) labeled by the ER lumenal marker GFP-HDEL are connecting ER (light green) and Golgi cisternae (light purple). Dynamic protrusions from Golgi bodies (purple) are labelled with the medial/*trans*-Golgi body marker ST-RFP. **(B)** Protrusions from ERES to ER (dark orange) can also be observed with the ERES/COPII components Sec24, Sec16 and Sar1a labelling a pre-*cis*-Golgi compartment (light orange).

Furthermore, fine ER tubules were seen to thread through Golgi bodies (**Figure 3A** time series, **Figure 3B**) and wrap themselves around Golgi bodies (**Figures 3A, C**). These thin tubules average about 20 nm diameter, considerably smaller compared to 40 nm diameter for the average ER tubule (Pain et al., 2019). Almost every Golgi body imaged appears to be attached to the ER *via* one or two of these ER-derived tubules. These tubules can also connect up to Golgi body protrusions (**Figure 3D**), but these are rare event to image and were only observed in approximately 1:1000 Golgi bodies. The morphology and location of these fine ER-derived and HDEL-labelled tubules distinguishes them from the tubules of the bulk ER network. To clearly refer to them, we termed them ERGo (ER-Golgi) tubules in the further text. A schematic representation of ERGo tubules and Golgi body protrusions is depicted in **Figure 4A**.

**Figure 5:**
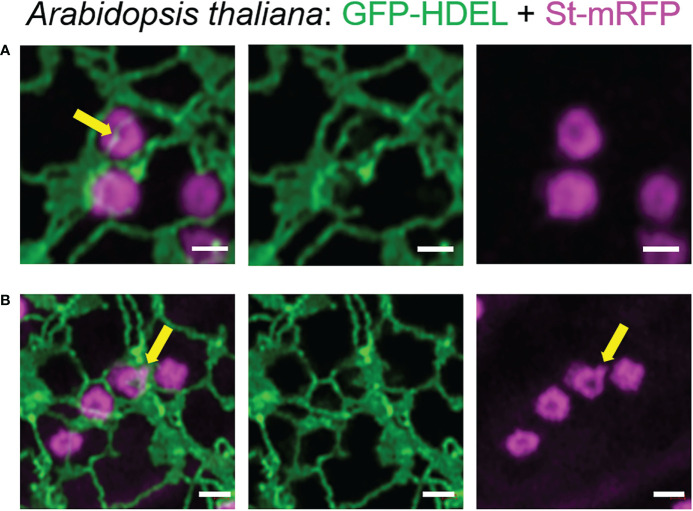
The organization of the ER and Golgi shown in tobacco can also be observed in Arabidopsis transgenic plants. ER tubules wrapped around and through the Golgi body (**A**, yellow arrow) and dynamic protrusions from the Golgi body (**B**, yellow arrow) were also observed in stably transformed *Arabidopsis thaliana* plants. Golgi bodies were labelled with the medial/*trans*-Golgi marker ST-mRFP (in magenta) and the ER is labelled with GFP-HDEL in green. Size bars = 1 µm.

To confirm that these tubules were not limited to transient expression in tobacco leaf cells, we also imaged the ER and Golgi body markers HDEL and ST in stable expression in *Arabidopsis thaliana* plants. Also in this system, the dynamic morphology with ERGo tubules (**Figure 5A**) and Golgi protrusions (**Figure 5B**) was observed (**Figure 5**; **Supplementary movie S2**).

**Figure 6:**
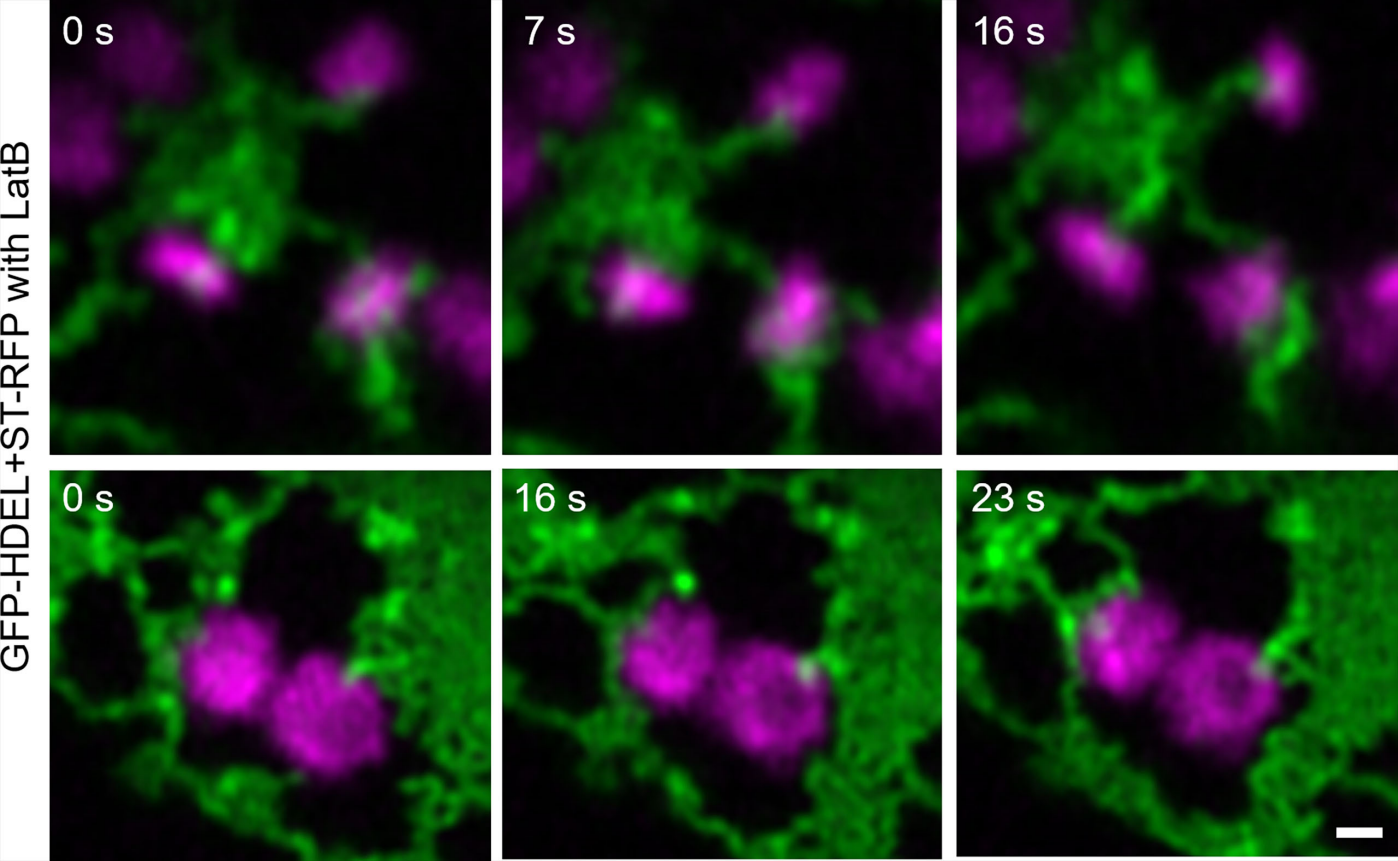
Changing ER dynamics with latrunculin B (LatB). The ER and Golgi markers GFP-HDEL (green) and ST-mRFP (magenta) are coexpressed in tobacco leaf epidermal cells, tobacco leaf pieces were treated with LatB and videos were taken. Time frames [s] are indicated. Size bars = 0.5 µm.

To investigate if ER dynamics impacts on ER-Golgi tubules, the drug latrunculin B (LatB) was applied (**Figure 6**). LatB inhibits actin polymerization and interrupts ER dynamics, resulting in immobile Golgi bodies. LatB treatment also resulted in the enhanced formation of ER cisternae (Pain et al., 2019). After LatB treatment, ERGo tubules were clearly visible but we could not determine if they remained through LatB treatment or were formed *de novo*. *De novo* formation of ERGo tubules would indicate that they are not only a physical connection required when ER and Golgi bodies are rapidly moving in the cell.

### Distribution of ER exit site markers and Golgi glycosylation enzymes

**Figure 7:**
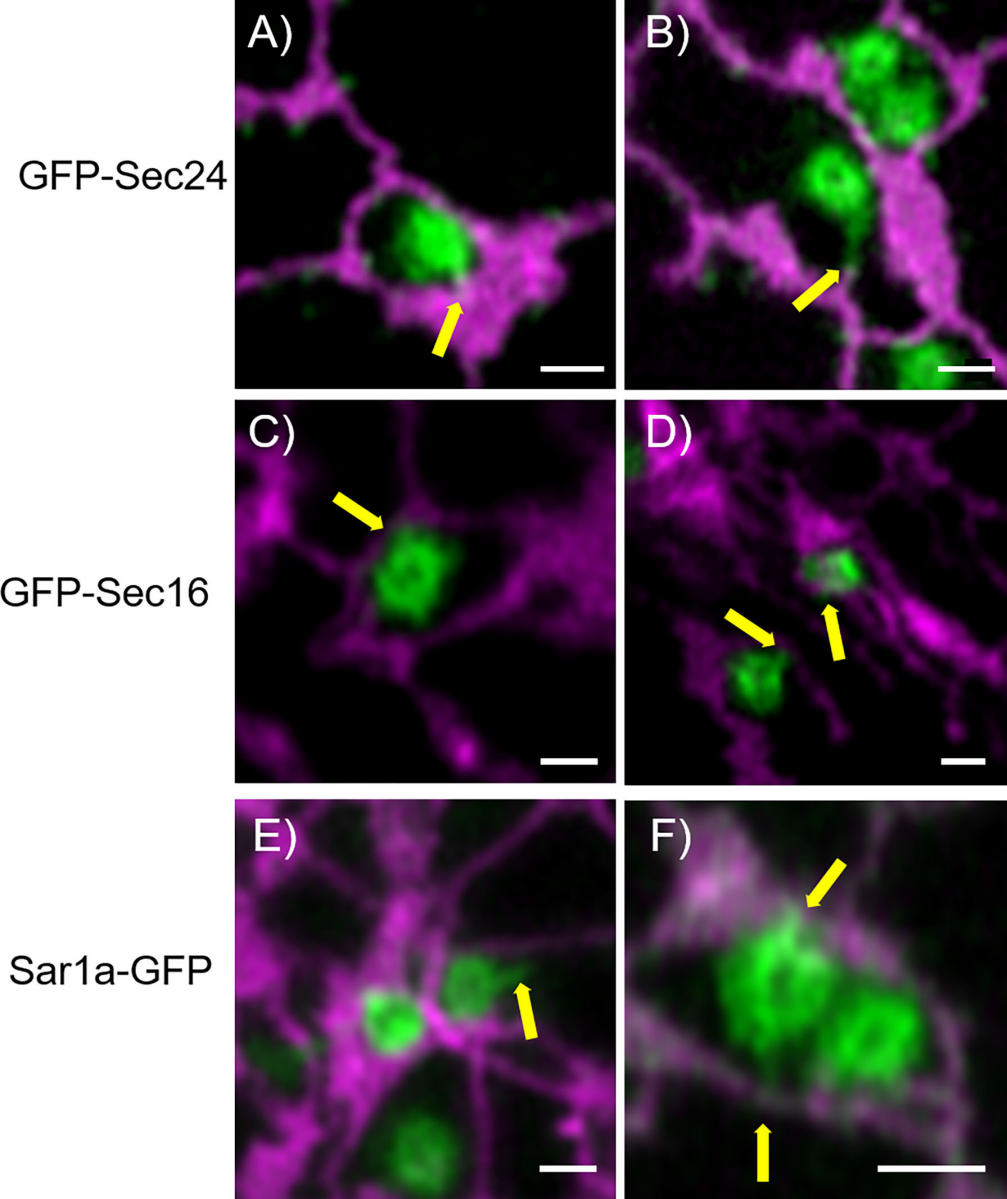
COPII components show protrusions that connect with the ER. The ERES markers/COPII components GFP-Sec24 **(A, B)**, GFP-Sec16 **(C, D)** and Sar1a-GFP **(E, F)** show dynamic protrusions (yellow arrows). Proteins are expressed transiently in tobacco leaf epidermal cells. The ER is labelled with the lumenal marker RFP-HDEL (magenta). Size bars = 1 µm.

To further investigate the physical link between ER and Golgi bodies, we wanted to look at a structure located between the ER and Golgi, the ERES. Interestingly, protrusions similar to those previously shown with Golgi markers (**Figures 2**, **5**) were also observed when ERES markers/COPII components were expressed together with the ER marker RFP-HDEL (Wang et al., 2014) (**Figures 4B**, **7**). We used functional fluorescent fusion markers of the COPII coat proteins GFP-Sec24 (Faso et al., 2009), GFP-Sec16 (Takagi et al., 2013) and Sar1a-GFP (Hanton et al., 2008). We found that the structures labelled with these markers showed dynamic protrusions in most cases wrapped around ER tubules or connecting with ER tubules (**Figure 7**) comparable to the protrusions labelled with the Golgi marker (**Figures 1**–**5**).

**Figure 8:**
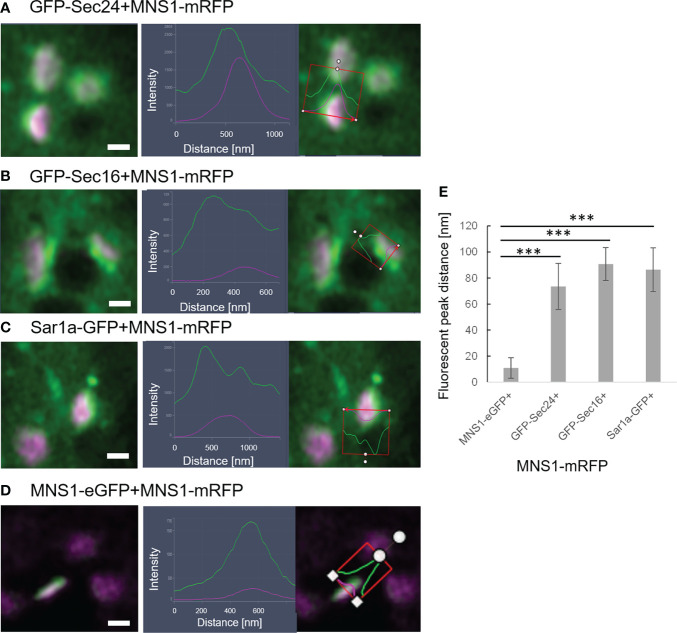
ERES markers define a pre-Golgi compartment. ERES markers GFP-Sec24 **(A)**, GFP-Sec16 **(B)** and Sar1a-GFP **(C)** are distributed closely to the *cis*-Golgi-marker MNS1-mRFP but do not fully overlap with MNS1-mRFP. Merged images in a side view are shown together with line profiles for both markers. Proteins were expressed transiently in tobacco leaf epidermal cells. Size bars = 0.5 µm. **(D)** MNS1-eGFP and MNS1-mRFP were coexpressed as a control for full colocalization. **(E)** Statistical analysis shows the distance [nm] between fluorescent peak of ERES marker and MNS1-mRFP. Significance was analyzed by Kruskal-Wallis (***p< 0.001). n = 3 with 5 technical replicas each.

It is also of great interest that ERES markers/COPII components did not label the ER as one would expect for ER vesicle budding sites but are closely and dynamically aligned with Golgi cisternae. We next aimed to gain deeper insights on the spatial relationship of the *cis*-Golgi and the COPII markers using high-resolution microscopy. When coexpressed with the *cis*-Golgi body marker MNS1 (Liebminger et al., 2009; Schoberer et al., 2014), the ERES markers GFP-Sec24 (**Figure 8A**), GFP-Sec16 (**Figure 8B**) and Sar1a-GFP (**Figure 8C**) labelled a compartment close to MNS1-mRFP, however their localization did not fully overlap (**Figure 8**). As a control, MNS1-mRFP was coexpressed with MNS1-eGFP (**Figure 8D**). The distance between the three tested ERES markers and the *cis*-Golgi marker MNS1 was statistically significantly larger than the distance between MNS1-mRFP and MNS1-eGFP (**Figure 8E**). This indicates that in plant cells the labelled ERES structures may represent a pre-*cis*-Golgi compartment with ERGo tubules and protrusions allowing for physical connections to the ER (**Figure 4B**).

**Figure 9:**
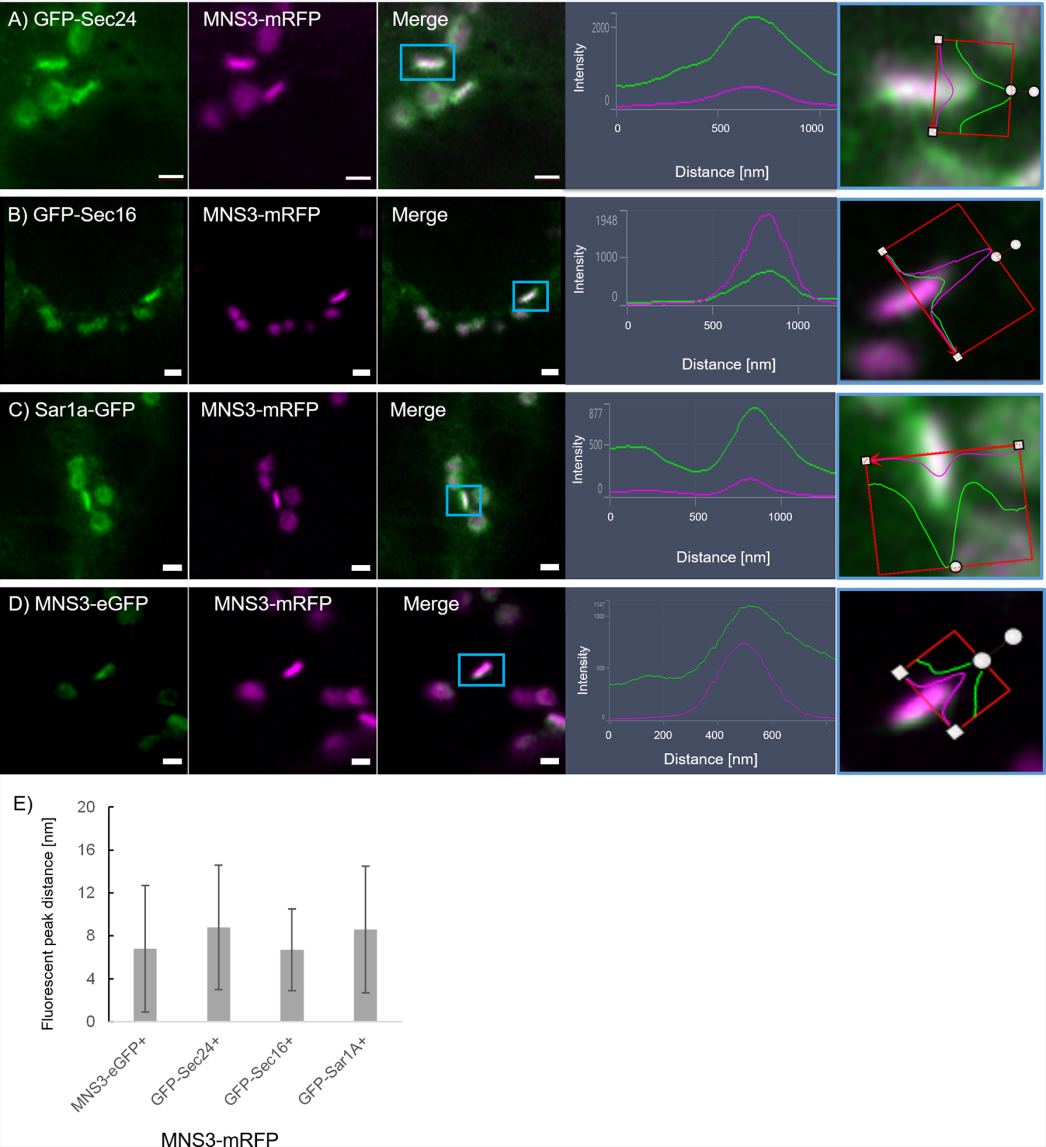
The ERES markers localize at the GECCO. ERES markers GFP-Sec24 **(A)**, GFP-Sec16 **(B)** and Sar1a-GFP **(C)** colocalize with MNS3-mRFP. **(D)** MNS3-eGFP and MNS3-mRFP are coexpressed as a control for full colocalization. Representative confocal images and analysis plots are shown for each combination. The blue box indicates the Golgi body shown enhanced on the right-hand side panels (merged images in side view together with line profiles for both markers). Proteins are expressed transiently in tobacco leaf epidermal cells. Size bars = 1 µm. **(E)** Statistical analysis shows the distance [nm] between fluorescent peak of ERES marker and MNS3-mRFP. Significance was analyzed by Kruskal-Wallis. n = 3 with 5 technical replicas each.

To further investigate a potential pre-*cis*-Golgi compartment, we coexpressed the ERES markers Sec24 (**Figure 9A**), Sec16 (**Figure 9B**) and Sar1a (**Figure 9C**) with MNS3, the Arabidopsis ER-α-mannosidase I (MNS3; (Schoberer et al., 2019)). MNS3 has previously been shown to partially colocalize with MNS1 and was suggested to reside in *cis*-most Golgi membranes, the Golgi entry core compartment (GECCO) (Ito et al., 2017). All tested ERES markers colocalized with MNS3 (**Figures 9A–C**). As a control, MNS3-mRFP was coexpressed with MNS3-eGFP (**Figure 9D**). The distance between the fluorescent peaks between ERES markers and MNS3 was analyzed and did not significantly differ from the control (**Figure 9E**). This confirms our results (**Figure 8**) of a presence of pre-*cis*-Golgi compartments with a specific composition of proteins such as the tested ERES/COPII proteins and MNS3.

### ERGo tubules are still present after inhibition of ER-to-Golgi transport

We next aimed to investigate a potential role for ERGo tubules in ER-Golgi transport. We analyzed the presence of the ERGo tubules in cells with inhibited ER-Golgi transport. Interfering with COPI coat assembly results in the inhibition of protein transport and a collapse of Golgi body membranes into the ER. The fungal metabolite brefeldin A (BFA) is a commonly used method to inhibit COPI coat formation. In plants, BFA targets a Sec7-type GEF that is necessary for activation of the small COPI GTPase Arf1p (Nebenführ et al., 2000). This leads to the dissociation of COPI coat proteins from Golgi membranes (Ritzenthaler et al., 2002) and the redistribution of Golgi markers, such as ST–GFP/RFP, to the ER and dilatation of the ER network (Boevink et al., 1998; Saint-Jore et al., 2002).

**Figure 10:**
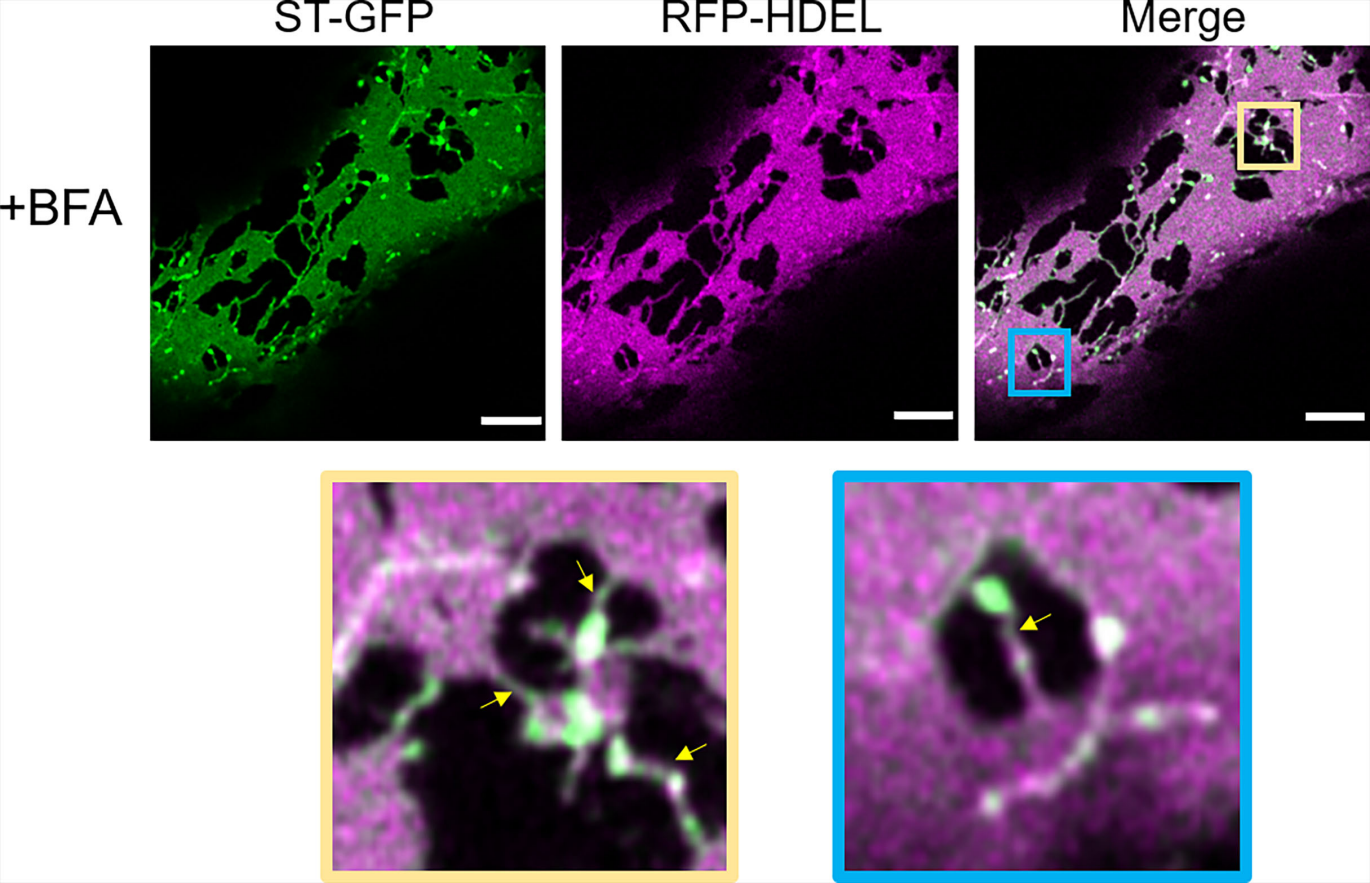
Disruption of COPI results in the redistribution of Golgi membranes into the ERGo tubules. The ER and Golgi markers RFP-HDEL (magenta) and ST-GFP (green) are coexpressed and the leaf pieces treated with BFA. Connecting tubules between ER and Golgi remnants are visible (yellow arrows). The blue and yellow boxes in the merge image indicate areas shown in higher magnification below. Size bars = 5 µm.

We aimed to use BFA to see if impairment of ER-Golgi transport would influence the presence of ERGo tubules and Golgi/ERES protrusions, which could be an indicator for a potential function of these structures in ER-Golgi transport. We found that BFA treatment on plants expressing ST-GFP and RFP-HDEL resulted in enhanced ER cisternae formation and as expected, redistribution of the Golgi marker ST-GFP to the ER (**Figure 10**). As typical for BFA treatment, small ST- and HDEL-labelled Golgi body remnants remained (Fujiwara et al., 1988; Driouich et al., 1993; Satiat-Jeunemaitre et al., 1996). Interestingly these remnants were connected to the ER by ERGo tubules and were labelled by both the ER and Golgi body markers RFP-HDEL and ST-GFP (**Figure 10**, zoom in yellow and blue frame). The distribution of ER and Golgi body marker appears not evenly distributed in places, which could be due to the inhibited transport. ERGo tubules were found in connection with all remnants of Golgi bodies (**Figure 10**). This suggests that the physical connection between ER and Golgi body remnants was maintained upon BFA treatment.

**Figure 11:**
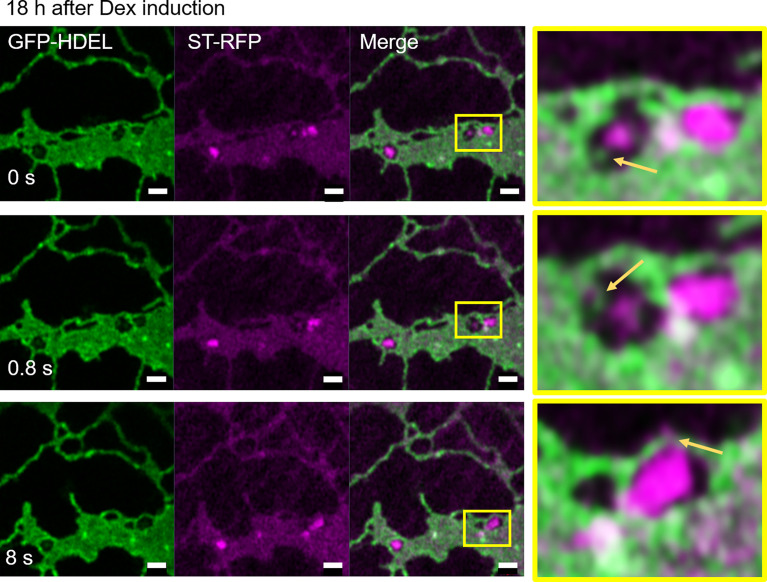
Disruption of Golgi membranes by inhibition of COPII uncoating leads to a segregation of ERGo tubules from Golgi remnants. The dominant negative mutant Sar1a-GTP is expressed in an inducible manner together with the ER and Golgi markers GFP-HDEL (green) and ST-mRFP (magenta). Imaging was carried out 18 h after dexamethasone (Dex) induction and a time-lapse (0 s, 0.8 s, 8 s) is shown. The yellow boxes in the images indicate areas shown in higher magnification on the right hand side. ERGo tubules and Golgi protrusions are indicated with yellow arrows. Size bars = 1 µm.

We next aimed to test the effect of blockage of the COPII pathway and test the hypothesis that a blockage of the progression of the COPII cycle with a dominant-negative mutant of the COPII GTPase Sar1a (Osterrieder et al., 2010) would allow to detect the sites of COPII assembly. We used Sar1a-GTP, a constitutively active (GTP-locked) mutant form of Sar1p from *Nicotiana tabacum*, which has been shown to inhibit protein transport between the ER and Golgi bodies (Andreeva et al., 1998; Takeuchi et al., 2000; Phillipson et al., 2001; daSilva et al., 2004; Hanton et al., 2008). Here a dexamethasone (Dex) inducible system in stably transformed *Nicotiana tabacum* plants allowing controlled expression of Sar1-GTP (Osterrieder et al., 2010) was applied. The ER and Golgi body markers, GFP-HDEL and ST-mRFP respectively, were expressed in these plants. We found that 18 h after Dex-induction, most of the ST-mRFP protein pool was located in the ER with only very few and smaller ST-labelled Golgi bodies remaining (**Figure 11**). Similar to BFA treatment, Sar1a-GTP expression resulted in enhanced ER cisternal areas, and ST-labelled Golgi stacks were quite tightly engulfed within ER lacunae. The stacks were again still connected to the ER by ERGo tubules but differently from with BFA treatment, the ERGo tubules only showed HDEL labelling with ST-labelled protrusions (**Figure 11**). ERGo tubules could only be seen in connection with remaining Golgi structures. Sar1a-GTP blocks the uncoating of the COPII coat and the Golgi marker is visible in association but still physically distinct from the ERGo tubules. These results, coupled with the findings that COPI inhibition leads to a redistribution of Golgi markers into the ERGo tubules, indicate that the ERGo tubules may be involved in the cargo transport to the Golgi body.

## Discussion

The nature of the ER exit sites together with ER-Golgi transport has been a source for discussion over the years. In yeast and mammalian systems COPII/COPI-dependent transport was the generally accepted mechanism, which was also transferred to plant systems.

In mammalian cells, ERES are morphologically recognized as areas on the rough ER membrane that are devoid of ribosomes and form coated buds, vesicles, and vesicular tubular clusters (Tang et al., 2005). These clusters undergo long-range transport mediated by microtubules to dock with the *cis*-face of the Golgi apparatus (Tang et al., 2005). ERES are characterized by the components of the COPII-coated membranes, v-SNARES required for docking and p24 proteins as cargo receptors (Strating and Martens, 2009). In yeast, COPII-coated vesicles have been suggested to bud from the ER membrane to transport cargo to very simple Golgi bodies that have the capacity to mature from *cis*- through to *trans*-forms (Matsuura-Tokita et al., 2006).

In Embryophyta, early conventional transmission electron microscopy did not show any visible coated ER areas or structures that could be convincingly inferred to be ERES. The application of ultra-rapid freezing techniques combined with tomographic electron microscopy though suggested that a COPII vesicle-mediated transport may exist (Kang and Staehelin, 2008). COPII components were indeed identified in plants using bioinformatics analysis (Robinson et al., 2007) but that does not exclude a non-vesicular localization. Live cell imaging methodologies using fluorescent protein markers showed that Golgi bodies are closely linked with the ER (Boevink et al., 1998; daSilva et al., 2004; Faso et al., 2009; Osterrieder et al., 2017). All of this supported the “secretory unit” concept stating that Golgi and ER exit sites are moving together with the ER (daSilva et al., 2004) and that Golgi biogenesis could be from newly generated exit sites (Hawes et al., 2008).

*In vitro* experiments using giant unilamellar membrane vesicles incubated with COPII components showed no vesicle formation but bead-like membrane organization (Bacia et al., 2011). Recent findings in mammalian systems also question the exclusivity of vesicular anterograde transport. Two research groups showed independently that carriers leaving the ER are not coated by COPII components and suggested the existence of different parallel carrier types including vesicles but also tubules, saccules and tunnels (Weigel et al., 2021; Shomron et al., 2021). In these studies, ERES expand into tubules, which contain cargo but no COPII components. These can only be found at the neck of the tubules; hence, they proposed that carriers leaving the ER are not COPII coated and that the role of COPII is to concentrate transport cargo into carriers (Weigel et al., 2021; Shomron et al., 2021).

Our analyses using a high-resolution confocal imaging approach have provided new insights into the organization of the ER and Golgi interface. By facilitating a visualization of the ER, COPII markers and Golgi membranes in living cells, we were able to capture structures and their dynamics in a manner that is not possible with ultra-resolution microscopy in fixed cells or cryo-electron tomography. Our analyses allowed us to follow the dynamics of the ERGo tubules, which appear to be thinner tubules compared to the bulk ER tubular network. We also showed that treatment with BFA causes a redistribution of Golgi markers into the bulk ER and the ERGo tubules with Golgi remnants distributed at the ERGo tubules, and that blockage of COPII with Sar1a-GTP leads to the formation of structures that are connected but not merged with ERGo tubules. It was previously suggested that the BFA-induced Golgi remnants contain a minimal set of *cis*-Golgi membrane proteins carrying out specific roles at the ER-Golgi interface, such as protein sorting, ER-Golgi tethering, and N-glycan processing. These BFA-induced structures could then be used as a reservoir to quickly produce *cis*-Golgi cisternae with materials directly from the ER (Hawes et al., 2010; Ito et al., 2012; Robinson et al., 2015; Schoberer et al., 2019), and accordingly, we propose ERGo tubular connections may provide physical stability between the ER and the Golgi as well as a rapid and targeted transport pathway.

Pre-*cis*-Golgi compartments have been previously suggested for proteins proposed to be involved in ER-Golgi tethering: AtGolgin-84A, which colocalizes with Sar1a (Vieira et al., 2020), as well as AtCASP (Latijnhouwers et al., 2007; Osterrieder et al., 2017) and the Arabidopsis ER-α-mannosidase I (MNS3; (Schoberer et al., 2019)). Our results support the existence of a pre-*cis*-Golgi compartment where COPII components accumulate rather than accumulating on the ER. The thin tubular connections, observed between ER and Golgi as well as between ER and ERES could be involved in cargo transport as well as in stabilizing the physical connection between ER and Golgi bodies. Although we have no evidence to infer a functional role to the ERGo tubules, the visualization of these new structures and the definition of their spatial distribution in relation to COPII markers and the visualization of a pre-Golgi compartment shed an interesting new light onto the ER-Golgi interface in plants and challenge the existing dogma on the existence of COPII vesicles in plants. So far, in plant systems no elegant ER-Golgi cargo transport assay such as the RUSH methodology (Boncompain et al., 2012) for mammalian systems has been established but this will be of great importance for studying an involvement of ERGo tubules in ER-Golgi transport.

## Data availability statement

The original contributions presented in the study are included in the article/**Supplementary Material**. Further inquiries can be directed to the corresponding author.

## Author contributions

VK, JS, and FB conceived the experiments. AMG, JS, CP, and VK carried out the experiments. JS, FB, and VK wrote the manuscript. All authors contributed to the article and approved the submitted version.

## Funding

This research was funded in part by the Austrian Science Fund (FWF) [P31921-B32] to JS. For the purpose of open access, the author has applied a CC BY public copyright license to any Author Accepted Manuscript version arising from this submission. FB is funded by MSU AgBioResearch [MICL02598]. VK is grateful for funding by the BBSRC [BB/W011166/1] and Oxford Brookes University. AMG is funded *via* the Oxford Interdisciplinary Bioscience Doctoral training Programme, CP is funded by Oxford Brookes University.

## Acknowledgments

The authors thank the Oxford Brookes University Centre for Bioimaging.

## Conflict of interest

The authors declare that the research was conducted in the absence of any commercial or financial relationships that could be construed as a potential conflict of interest.

## Publisher’s note

All claims expressed in this article are solely those of the authors and do not necessarily represent those of their affiliated organizations, or those of the publisher, the editors and the reviewers. Any product that may be evaluated in this article, or claim that may be made by its manufacturer, is not guaranteed or endorsed by the publisher.

## Supplementary material

Supplementary Figure 1 Video tobacco transiently expressing GFP-HDEL and ST-mRFP.

Supplementary Figure 2
**(A, B)** Videos Arabidopsis stably transformed with GFP-HDEL and ST-mRFP.
